# Supplementary material for: Assessing the Safety and Therapeutic Efficacy of Cannabidiol Lipid Nanoparticles in Alleviating Metabolic and Memory Impairments and Hippocampal Histopathological Changes in Diabetic Parkinson’s Rats
Source: Pharmaceutics. 2024 Apr 8;16(4):514. doi: 10.3390/pharmaceutics16040514 (PMC11054774; doi:10.3390/pharmaceutics16040514)
Supplement: Supplementary file 1 [file pharmaceutics-16-00514-s001.zip › pharmaceutics-2889126-supplementary.pdf]

Supplemental data

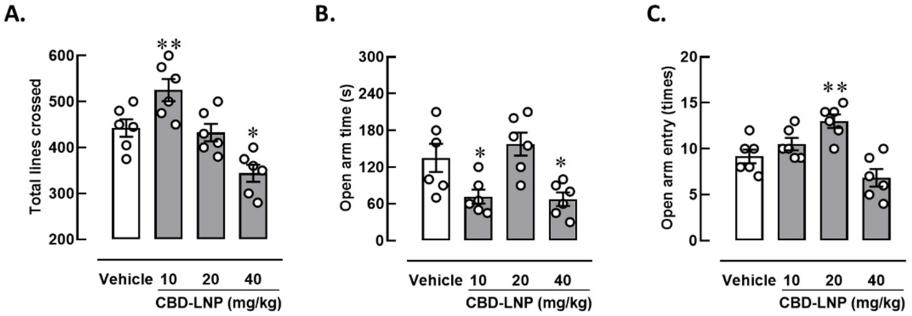

Supplementary Figure S1. Effects of CBD-LNP on locomotor activity and anxiety-like behaviors in normal male rats. (A) Number of total line crossed in the open filed test (B) open arm entry and (C) open arm time in the elevated-plus maze. Data are presented as mean ± SEM (n=6 rats/group). **P* < 0.05, ***P* < 0.01 compared to vehicle-treated control group.

Supplementary Table S1. Metabolic parameters at baseline and after diabetic induction

Metabolic parameters	Control group			Diabetic group		
	**Baseline**	**Fed normal food diet**	**0.01 M sodium citrate injection**	**Baseline**	**Pre-treatment with high fat diet**	**STZ injection**
	9-week-old	10-week-old	11-week-old	9-week-old	10-week-old	11-week-old
Body weight (g)	228.00±4.01	250.75±5.10	283.63±2.21	231.13±3.56	263.50±6.86	300.00±4.33**
Triglycerides (mg/dL)	89.88±2.97	89.13±4.65	90.88±2.84	88.13±3.89	90.00±2.84	124.00±6.74***
Total cholesterol (mg/dL)	74.38±3.95	76.88±4.00	79.13±3.02	75.00±2.67	77.25±3.42	139.38±16.41**
Fasting blood glucose (mg/dL)	98.75±3.98	105.63±2.49	107.50±5.26	98.13±2.49	106.88±3.89	243.38±17.97***
Insulin (µU/L)	29.23±1.01	29.60±1.07	29.35±0.98	29.12±1.06	29.15±1.68	22.22±1.99**
HOMA-IR index	7.35±0.42	7.50±0.36	7.38±0.34	7.44±0.27	7.68±0.56	12.59±1.25***
HOMA-β index	284.13±18.89	281.69±24.01	283.60±24.64	282.74±22.53	246.69±15.10	51.05±6.65***

Data are presented as mean ± SEM (n=8 rats/group). HOMA-IR (Homeostatic Model Assessment for insulin resistance), HOMA-β (Homeostatic Model Assessment estimates steady state beta cell function). **P < 0.01, *** < 0.001 compared to baseline.
